# Learning-based parallel acceleration for HaplotypeCaller

**DOI:** 10.1186/s12859-025-06242-w

**Published:** 2025-08-20

**Authors:** Xiangxing Lai, Minguang Xiao, Lingling Weng, Zhiguang Chen

**Affiliations:** 1 School of Computer Science and Engineering, Sun Yat-sen University, Waihuan Dong Road, Guangzhou, 510006 China; 2 National Supercomputer Center in Guangzhou, Xiaoyuan Dong Road, Guangzhou, 510330 China

**Keywords:** HaplotypeCaller, Learning, Parallel, Acceleration

## Abstract

In the genome analysis workflow, Genome Analysis Toolkit (GATK) HaplotypeCaller is a widely used variant calling tool designed to accurately identify single nucleotide polymorphisms (SNPs) and insertions/deletions (Indels) in samples. However, when processing large-scale datasets, HaplotypeCaller often faces the challenge of excessively long runtime. Parallelizing GATK HaplotypeCaller with data segmentation is an effective solution, but existing methods struggle to accurately estimate the computational complexity of each data block, leading to severe computational skew. This paper introduces a learning-based framework LPA (learning-based parallel acceleration), leveraging model to accurately predict the computational complexity of data. By employing adaptive data segmentation algorithms and Multi-Knapsack Problem (MKP) based task scheduling, LPA significantly alleviates computational skew. We evaluated LPA in multiple datasets, demonstrating that its execution speed is 30x–40x faster than HaplotypeCaller and 2x–5x faster than HaplotypeCallerSpark. LPA achieves a speedup of 1.3x–2x compared to similar methods. And LPA maintaining a high accuracy with over 99.9%, enhancing the efficiency and reliability of variant calling. The source code of LPA is publicly available at https://github.com/laixx9/LPA.

## Introduction

### Overview

With the continuous advancement of sequencing technologies, the limitations of GATK have become increasingly apparent. This section first outlines the inefficiencies in GATK’s computational performance, particularly the issue of load imbalance in its Spark-based implementation (GATK’s Limitations) section. We then review existing optimization strategies and analyze their strengths and limitations (Optimization Strategies) section. To address these problems, We propose LPA, an Artificial Intelligence(AI)-based approach that alleviates load imbalance and boosts performance (Learning-based Parallel Acceleration) section.

### GATK’s limitations

**Fig. 1:**
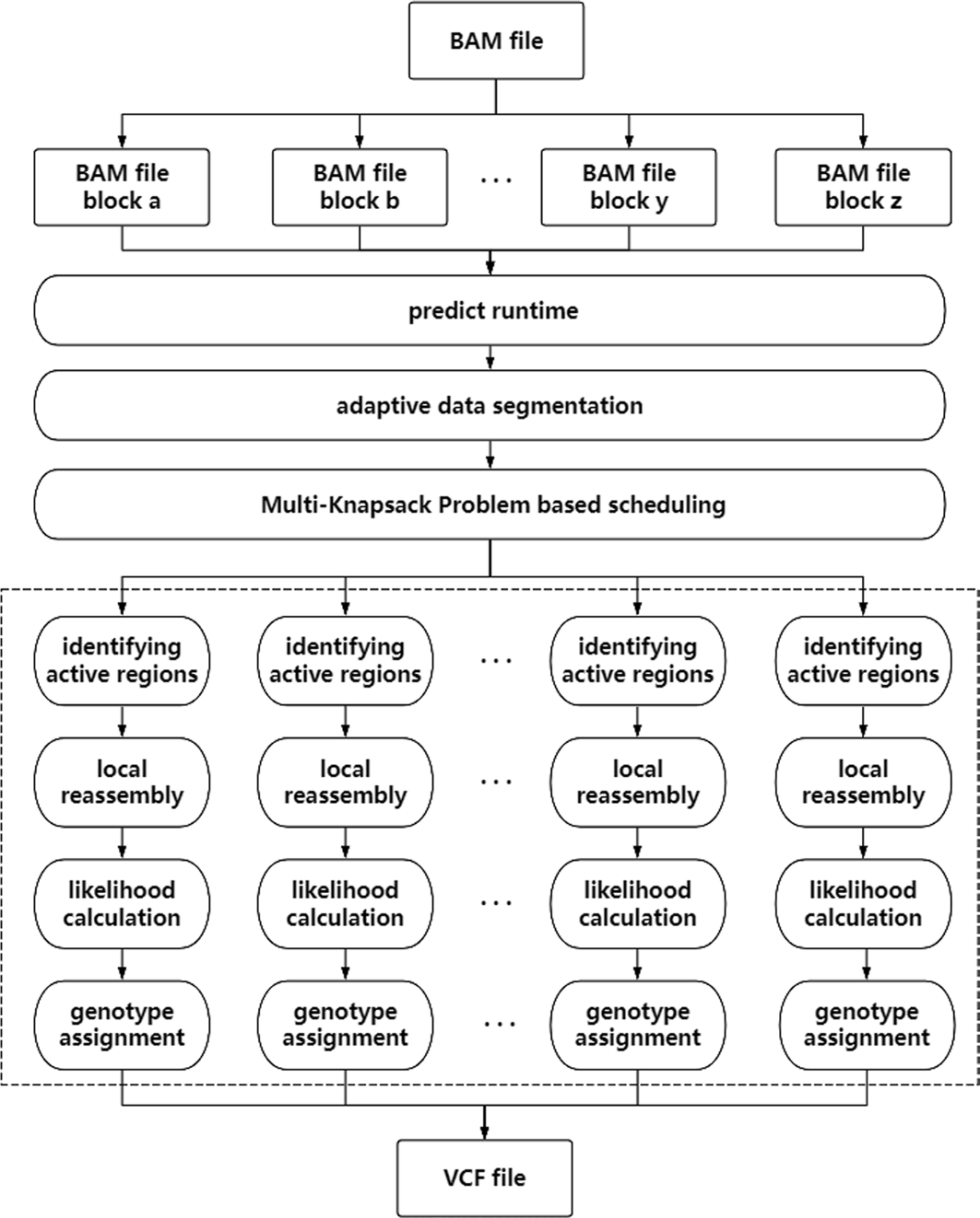
Overview of LPA

Driven by the technological advancement in recent years, next-generation sequencing(NGS) has become the cost-effective and widely adopted method for diagnosing genetic diseases [3, 7, 11, 16]. However, NGS generates large volumes of sequencing data, and the efficient analysis to so large volumes of data is a great challenge in the bioinformatic area. Accordingly, a variety of tools have been developed to accelerate the gene data processing. Among these tools, while some evaluations [9, 10] have noted that GATK has certain limitations in variant calling, GATK HaplotypeCaller remains the de facto standard software, widely adopted across numerous applications. However, the state-of-the-art HaplotypeCaller is known to suffer from low computing-efficiency due to its limited parallelism, imbalanced workloads, irregular data accesses, etc. Although GATK 4.0 introduced a multi-threaded version, HaplotypeCallerSpark, it suffers from severe computational skew [6], leading to load imbalance. To investigate the load imbalance issue of HaplotypeCallerSpark, we conducted the following experiment by analyzing the runtime distribution of its task partitions. As shown in Fig. 1, the majority of tasks completed within 0–20 min. However, there exist some outlier tasks whose runtime can extend up to 150 min. This implies that when other threads finish their tasks, they must wait for these long-running tasks to complete. With 64 threads, this results in a waiting time of 30–80 min for the other threads, which reduces resource utilization efficiency. Furthermore, as the number of threads increases, the overall runtime does not decrease due to the presence of these long-running tasks, which significantly limits its scalability.

**Table 1:** Distribution of data block runtime across datasets

Dataset	Runtime range (min)							
	0–20	20–40	40–60	60–80	80–100	100–120	120–140	140–160
HG00101	494	5	3	0	0	1	0	1
HG01256	452	6	2	1	0	0	1	1
NA12878	321	3	0	0	1	1	0	0
NA18561	453	9	0	0	2	0	0	0

### Optimization strategies

In recent years, several optimization methods for HaplotypeCaller have been proposed. Some superior approaches [12, 18, 21, 22, 25] employ graphics processing unit(GPU) or field-programmable gate array(FPGA) to parallelize computing-intensive algorithms (e.g., PairHMM) involved in HaplotypeCaller, significantly improving performance in critical steps of variant calling. These approaches achieve remarkable speedups by exploiting the high parallelism of domain-specific hardware, whereas the dedicated hardware indicates that these solutions are not cost-effective and hard to be deployed into the general-purpose computing platforms.

Furthermore, reconstructing the entire pipeline is an efficient optimization strategy. OVarFlow [4], for example, has automated the HaplotypeCaller process, significantly improving computational resource utilization. snpArcher [13] provides a standardized variant calling pipeline, which contribute to a broader understanding of genetic variation across species by facilitating the rapid use and reuse of large genomic data sets. Some studies have focused on reconstructing GATK to enhance performance. For example, LUSH [24] reimplemented GATK using C/C++ and introduced a new parallel framework, making the overall pipeline approximately 17 times faster than the standard GATK. Additionally, leveraging the Apache Arrow framework to process SAM format data in memory has also effectively improved resource utilization [1]. In addition to performance optimization, considerable research has focused on improving the accuracy of variant detection. For example, Jenever [19] employed a standard Transformer encoder with a dual-decoder architecture, achieving the highest F1 score in single nucleotide variant (SNV) calling. Karamoko Niaré et al. [17] optimized the variant calling pipeline for Plasmodium falciparum based on GATK4, significantly enhancing the sensitivity of variant calling.

As the Hadoop and Spark have been extensively adopted to parallelize large-scale data processing, using them to speedup HaplotypeCaller is a straightforward way to accelerate gene analysis. Spark, combining high-speed distributed computing with robust fault tolerance, provides a reliable foundation for this integration. Its resilience relies on RDD lineage (recomputing lost data via dependencies), task retries with node blacklisting (handling transient failures), and checkpointing (safeguarding against corruption), ensuring stable execution amid parallel processing issues. This blend of speed and reliability makes Spark ideal for accelerating gene analysis through HaplotypeCaller integration, balancing efficiency and robustness. Thus, many scholars, including us, use Spark as GATK’s parallel framework [2, 14, 15, 26]. For instance, SparkGA [15] divides input sequencing data into equally sized chunks and uses Spark to parallelize the entire genomic analysis workflow. This approach significantly accelerates variant calling, however, it faces severe long-tail latency when running HaplotypeCaller in parallel. The primary cause of long-tail latency is attributed to the varied computational complexity of sequence data, where even though the sequence data is divided into equally sized chunks, the latency of processing each of the chunks varies significantly.

To address this issue, ADS-HCSpark [26] proposed a method to alleviate computational skew by predicting the computational complexity of sequence data. This approach estimates computational complexity based on metrics such as the number of variants and reads, and accordingly splits high-complexity data blocks into much smaller chunks further. The fine-grained data segmentation and scheduling help to alleviate computational skew, but still suffers from the low estimation accuracy, since that computational complexity is influenced by a variety of metrics, rather than just the metrics considered by ADS-HCSpark. The misprediction may even exacerbate the computational skew.

### Learning-based parallel acceleration

In the past decade, AI technologies have attracted much attention and played critical roles in a variety of application areas. We found that AI model can be used to predict the computational complexity of sequence data as well, and the prediction accuracy could be improved significantly under well-designed models. Based on the above observation, in this work, we propose a high performance framework, which resorts to the AI model to generate balanced workloads for the parallel tasks executing HaplotypeCaller, thus significantly reducing the long-tail latency. Contributions of our work are concluded as follows:

- We build a dataset(https://github.com/laixx9/LPA-dataset) as well as train an AI model, which is able to accurately predict the computational complexity of sequence data. Based on the prediction of the AI model, we introduce an adaptive data segmentation algorithm which tries the best to generate data segments that have similar computing overhead.
- We employ the Multi-Knapsack Problem (MKP) to optimize the scheduling of data segments processing. Specifically, as each data segment corresponds to a latency estimated by the AI model, we group these data segments into several batches according to the estimated latencies, guaranteeing that total latency of processing each batch is approximately the same.
- We implement the aforementioned algorithm and policy in Spark, and evaluate the performance via a large range of datasets. The experimental results demonstrate that LPA outperforms other methods in not only speed but resource utilization.

## Methods

### Overview

This work proposes LPA, a learning-based framework that leverages model to accurately predict the computational complexity of data and employ adaptive data segmentation algorithms and task scheduling to alleviate computational skew. The entire workflow of LPA is illustrated in the Fig. 1. We trained an AI model using the collected data and leveraged this pretrained model to predict the computational complexity of each data block (Model) section. Since computational complexity is difficult to quantify directly, we use execution time as a proxy. After obtaining the estimated execution time for each block, we apply our adaptive segmentation algorithm to split blocks with longer runtime (Adaptive segmentation algorithm) section. Since task runtimes are known, multi-threaded task scheduling problem is closely resembles a Multi-Knapsack Problem , we adopt MKP-solving methods as our scheduling algorithm (Multi-Knapsack problem based scheduling) section. Finally, we execute the HaplotypeCaller workflow.

### Model

The HaplotypeCaller workflow typically consists of four steps: identifying active regions, local reassembly, likelihood calculation, and genotype assignment. Among these, the likelihood calculation stage accounts for approximately 70% [8] of the total runtime, with the PairHMM algorithm being the critical component. The time complexity of PairHMM is O (N * M * R * H) [8], where N is the number of reads, M is the number of candidate haplotypes, R is the total length of reads, and H is the total length of candidate haplotypes. However, these parameters cannot be directly obtained from the raw BAM file and require the completion of the first two stages to determine their values. This dependency significantly increases preprocessing time, making it impractical to predict PairHMM runtime directly from these metrics. To address this, we use related information from the data to estimate the computational complexity of PairHMM. The number of candidate haplotypes is generally correlated with the number of variants in an active region, while the total lengths of reads and haplotypes are linked to the depth of coverage in the active region. Thus, the runtime of PairHMM is primarily influenced by factors such as the number of variants, the number of reads, and the depth of coverage.

In addition, through our observations we have found that for chromosome 16, in the interval of 33,000,000 to 47,000,000, the runtime is significantly longer. In our dataset, this region occupies 73 positions in the top 100 longest runtime, while other related data (such as the number of variants or coverage depth) does not show significant differences. This suggests that genomic position may also affect runtime, potentially due to more complex variants in certain reference genome regions. This leads to substantial runtime differences for the same data under different positions. Additionally, the quality score of each read can, along with the number of variants, influence the identification of active regions. Therefore, as shown in Table 2, we use these metrics as features in the model to analyze and optimize computational efficiency.

In LPA, we employ the CatBoost [20] algorithm to predict runtime, a high-performance machine learning algorithm developed by Yandex based on Gradient Boosting Decision Trees (GBDT). CatBoost is highly effective in handling categorical features and missing values and is widely applicable to tasks such as regression, classification, multi-class classification, and ranking. The algorithm is known for its high training speed and low memory consumption, making it highly efficient. Furthermore, CatBoost offers excellent portability, enabling efficient execution on both ARM and x86 architectures. It also provides robust Java support, allowing pre-trained models to be directly utilized in Java environments without additional steps. This simplifies the deployment process, prevents data leakage during training, and ensures that trained models can be easily deployed across different machines by simply transferring the model, significantly enhancing cross-platform usability and security.

**Table 2:** Feature descriptions

Feature	Description
Chromosome	Identifies the specific chromosome
Start position	Starting point of the block on the chromosome
End position	Ending point of the block on the chromosome
Block number	Identifier for a specific data block
Length	Length of the block (in base pairs)
Number of reads	Number of reads in this block
Total quality score	Sum of quality scores in this block
Total variants	Sum of variants in this block
Sum of quality scores * Variants count	Sum of each read’s quality scores multiplied by variants count
Variant density	Number of variants per position
Coverage	Average number of reads covering each position
Reference genome	Version of the reference genome used
Time	Runtime of the block

### Adaptive segmentation algorithm

In cases of extreme computational skew, a single task’s runtime may dominate the total process time. Thus, we propose an adaptive data segmentation algorithm. This method targets the top n data blocks with the longest predicted runtime and segments them into smaller sub-blocks. Since these blocks usually contain a large number of reads, the impact of segmentation on final results is negligible. The number of segmentation is flexible, with a default setting of 8 sub-blocks, significantly shortening individual task runtime and reducing the overall process time.

The number of n is determined by a combination of a fixed percentage and a dynamic floating value. Based on extensive data observations, the top 8–10% of data blocks have the greatest impact on results. Therefore, we set a fixed value of 9% to handle most scenarios effectively. In general, segmenting the top 9% of blocks achieves the desired performance. However, for some datasets with a large number of long-running blocks, relying solely on a fixed 9% is insufficient. To address this, we introduce a floating value representing the number of blocks with runtime exceeding a certain threshold, as defined in Algorithm 1. As the size of input data increases, the threshold runtime for blocks significantly affecting results also rises. To adapt, we categorize the input data based on its size, and each level having a different threshold value. The final number of blocks to segment is determined by the maximum of tasks number * 9% and the floating value. This ensures that long-running tasks are handled in a timely manner, reducing bottlenecks caused by individual tasks and improving the stability and adaptability of the algorithm. By employing this adaptive segmentation strategy, we significantly reduce runtime and enhance overall computational efficiency.

Algorithm 1. Adaptive Segmentation Algorithm
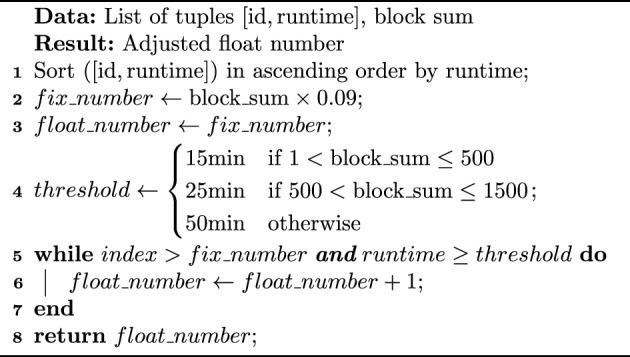


### Multi-Knapsack problem based scheduling

In the task scheduling problem, our goal is to allocate N tasks with known runtime to K threads (or computing nodes) in a balanced manner, aiming to minimize the overall runtime. This problem can be modeled as a MKP and solved using Dynamic Programming (DP). In this model, items correspond to tasks, the weight of an item corresponds to the runtime of a task, knapsacks correspond to threads or computing nodes, and the capacity of a knapsack corresponds to the maximum allowable load (total runtime) of a thread. Finding a load-balanced scheduling solution essentially involves determining the smallest knapsack capacity that can accommodate all tasks, thereby minimizing the overall runtime. As shown in the Algorithm 2, the time complexity of this problem is *O*($$N*K*I$$), where N is the number of tasks, K is the thread count and I is the iteration count. However, in our situation, we can further reduce the complexity through optimization. First, the lower bound of T is the maximum runtime of a single task, which provides a clear starting point for the search range. Second, since we only need an approximate optimal solution, we can limit the possible values of T by adjusting the incremental step size. Typically, the algorithm can find the optimal solution within five iterations, thereby reducing the time complexity to *O*($$5*N*K$$). This optimization makes the algorithm extremely efficient, with its execution time having almost negligible impact on the overall scheduling results. Therefore, this scheduling method is not only highly efficient but also demonstrates significant performance advantages in LPA.

Algorithm 2. Multi-Knapsack Model Based Scheduling Algorithm
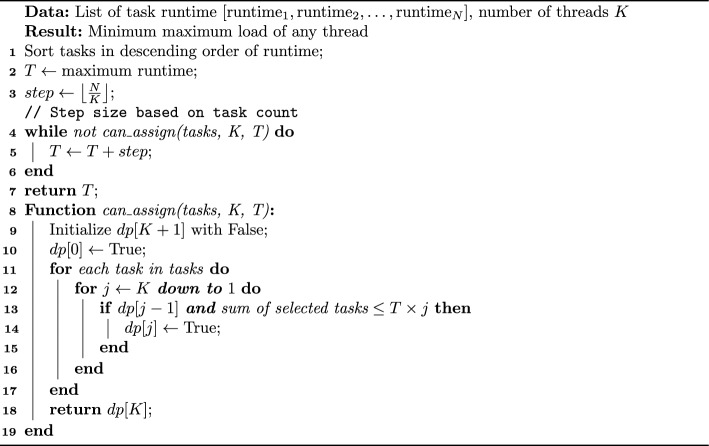


## Evaluation

### Overview

In this section, we evaluate LPA. We begin by describing the experimental setup and the datasets used for evaluation (Setup and datasets) section. We then present performance metrics, including runtime efficiency on both ARM and x86 architectures and scalability (Performance metrics) section. In addition, we assess the effectiveness of each component of LPA (Effectiveness of component) section and conclude with an evaluation of several system-level metrics (System metrics) section.

### Setup and datasets

We evaluated LPA on Kunpeng 920 server, which equipped with 128 core and 251GB memory. The GATK version is 3.8, and the Spark version is 3.6.

The training data is publicly available datasets in the 1000 Genomes Project [5, 23]. A total of approximately 300 datasets are used, amounting to 3.07 TB of data and containing 96561 records. The reference genome for all datasets is GRCh38_full_analysis_set_plus_decoy_hla.fa.

We use 6 datasets to evaluate LPA, a small dataset HG00281, four normal-sized datasets HG00101, HG01256, NA12878 and NA18561. To evaluate the model’s generalization capability, we add a larger high-coverage dataset NA18525. additionally. The reference genome for these six datasets is GRCh38_full_analysis_set_plus_decoy_hla.fa. Detailed information about these datasets is presented in Table 3.

**Table 3:** The detail information of five datasets

Sample ID	Population origin	Cell source	Application
HG00101	Great Britain (GBR)	EBV-transformed lymphoid	ChIP-seq and other omics studies
HG00281	Finnish (FIN)	Lymphoblastoid	SNV/Indel calling
HG01256	Colombian (CLM)	EBV-transformed cell line	WGS, CNV analysis
NA12878	European ancestry(CEU)	Blood/CEPH cell line	Gold-standard sample
NA18561	Han Chinese(CHB)	Lymphoblastoid	Phase 3 SNV/Indel variant calling

### Performance metrics

#### Runtime comparison

**Fig. 2:**
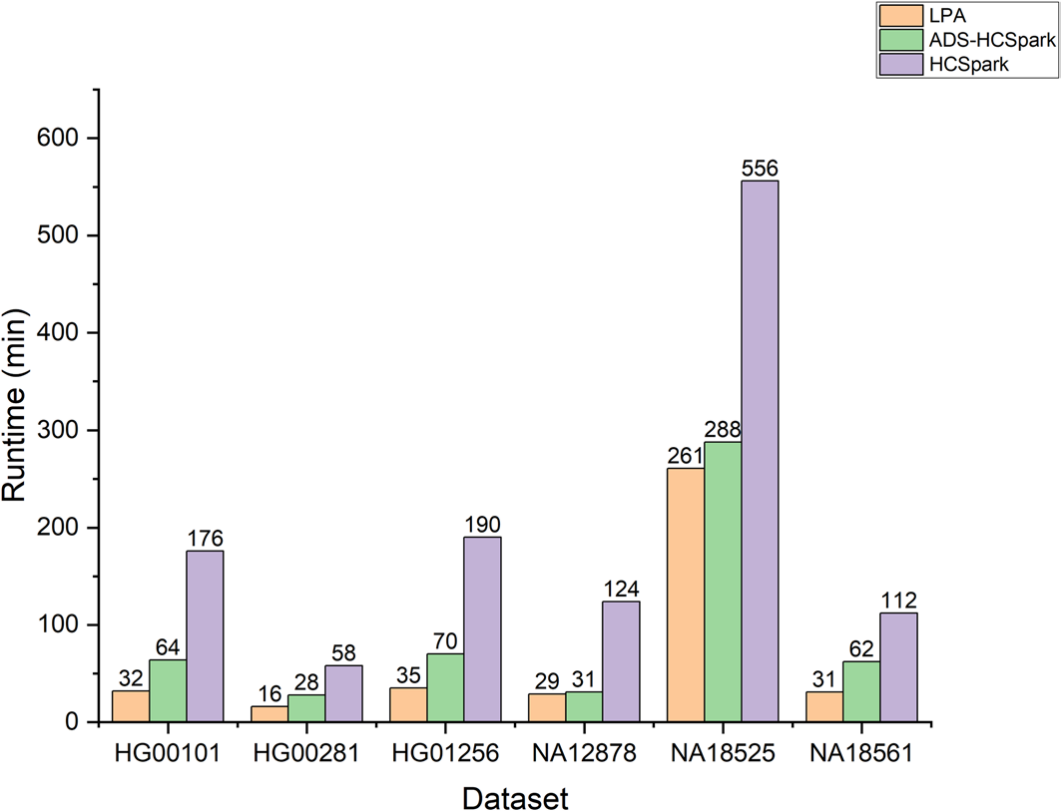
Performance comparison of three methods

We evaluated the performance of LPA and compared it with ADS-HCSpark and HaplotypeCallerSpark. The experiments were conducted in a 64-thread environment, and the results are shown in Fig. 2. LPA demonstrated superior performance across all six datasets compared to the other two methods. HaplotypeCallerSpark performed the worst due to severe computational skew, which caused long-tail delays and limited its overall performance. In the figure, we use HCSpark to refer to GATK HaplotypeCallerSpark, and this naming is used consistently throughout the subsequent figures.

While ADS-HCSpark partially alleviated this issue, it still exhibited shortcomings. In the HG00101 and NA18561 datasets, ADS-HCSpark failed to detect two tasks with long execution times (over 40 min), leading to an increase in the overall runtime by approximately 30 min. In the HG00281 dataset, although ADS-HCSpark successfully identified long-running tasks, the unordered nature of its set placement caused a 16-minute task to execute after other long-running tasks, resulting in an 10-minute delay.

In contrast, LPA, leveraging learning-based prediction and task prioritization, effectively addressed these issues and avoided long-tail delays. Notably, in the NA12878 dataset, the performance of LPA over ADS-HCSpark was similar. This was because both methods identified the task with a runtime exceeding 30 min, but the task continued running after all other tasks had completed, becoming a bottleneck for further performance improvement. As a result, the performance of LPA and ADS-HCSpark in this dataset was similar. Although we didn’t use high coverage data to train the model, LPA still perform well in high coverage dataset NA18525. This show that the model has good generalization capability.

The composition of LPA’s total running time is shown in Table 4. Notably, preprocessing time accounts for less than 5% of the total running time, indicating that LPA’s prediction process is highly efficient and has a negligible impact on the overall runtime.

**Table 4:** Composition of LPA’s total running time(second)

Dataset	Preprocessing time	Runtime	Total runtime
HG00101	47	1912.6	1959.6
HG00281	39	973.8	1012.8
HG01256	52	2078	2130
NA12878	42	1725.6	1767.6
NA18561	50	1816.6	1866.6

The performance results of the three methods on other datasets are shown in the Table 5. Due to time constraints, some datasets were not executed on HCSpark, so the time values for unexecuted datasets are set to empty.

**Table 5:** Performance comparison of three methods on other datasets (min)

Dataset	LPA	ADS-HCSpark	HCSpark
HG04195	51.98	81.27	130.00
HG06984	58.62	58.32	120.00
NA18531	57.08	55.68	195.00
HG01112	66.15	97.92	112.00
NA20868	38.17	58.55	125.82
HG01119	31.97	37.18	
HG01121	71.60	83.50	
NA19652	40.75	64.83	
HG00436	63.83	77.90	

**Fig. 3:**
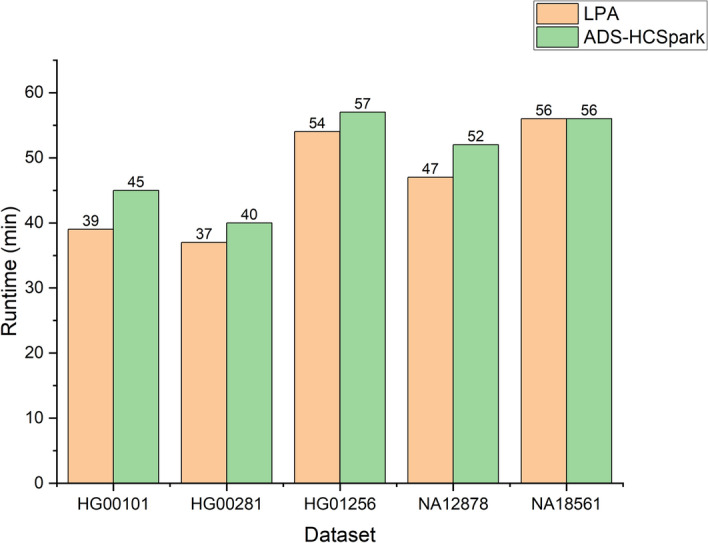
Performance comparison of two methods under x86 architecture

To investigate the performance of LPA under different CPU architectures, we conducted experiments with 32 threads on an x86 architecture. The results of LPA and ADS-HCSpark on five datasets are shown in the Fig. 3 LPA significantly outperforms ADS-HCSpark across 4 datasets, attributed to its more accurate prediction model and optimized scheduling strategy. Notably, the performance gain of LPA over ADS-HCSpark is less pronounced on the ARM architecture. Analysis suggests this may result from GATK’s targeted optimizations for x86, hardware configuration differences between the two servers, which alter the time proportion of each processing stage and thus the weight of features in the results. Additionally, since LPA’s training data were collected from ARM servers, its prediction accuracy decreases slightly on x86. Despite this, LPA still demonstrates superior performance compared to ADS-HCSpark.

#### Scalability

**Fig. 4:**
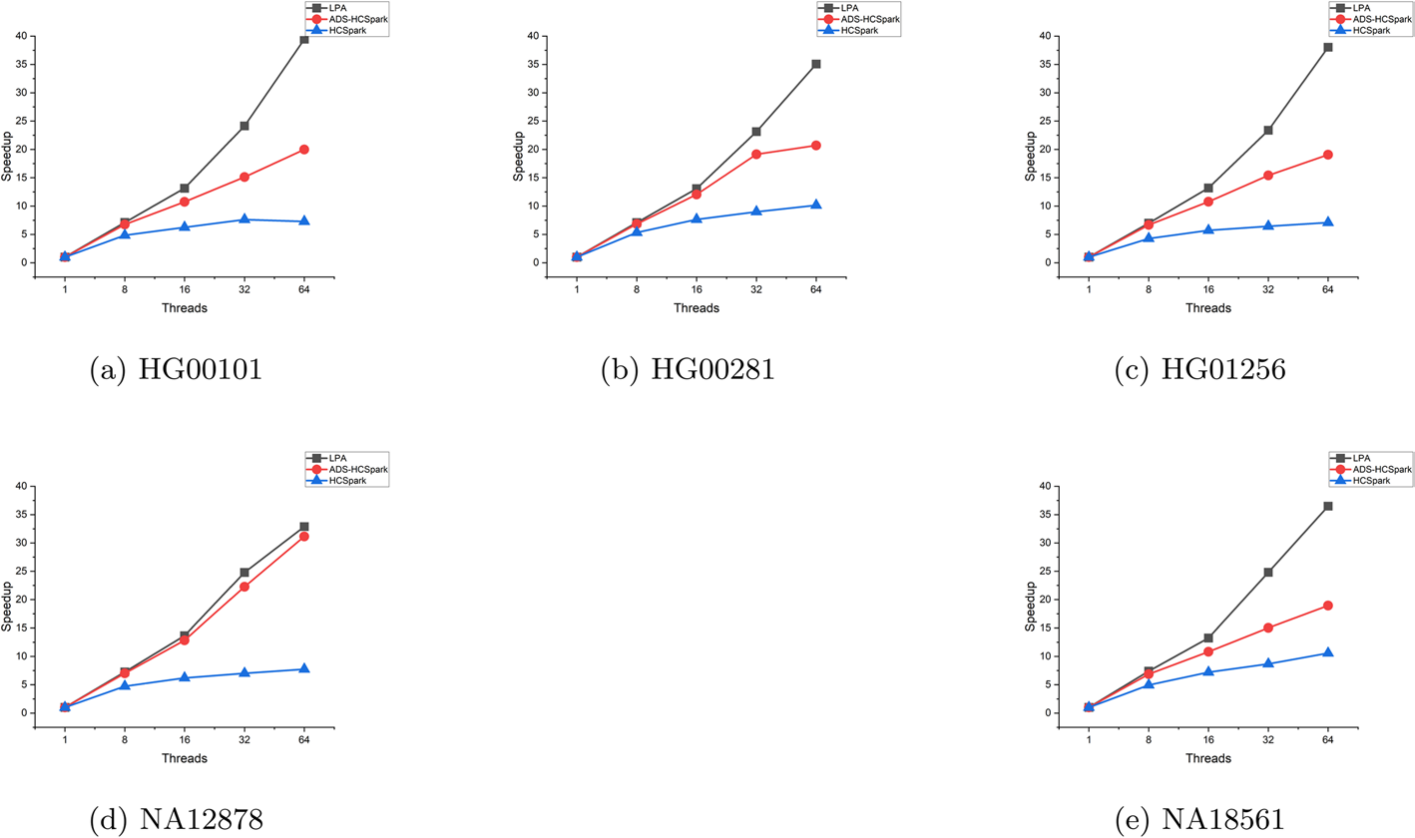
Comparison of speedup for three methods on various datasets

The performance of HaplotypeCaller does not necessarily scale with an increasing number of CPU cores. Its performance is constrained by various bottlenecks, including contention for shared resources such as cache and memory, as well as communication overhead. We conducted experiments to evaluate the scalability of LPA and compared it with ADS-HCSpark and HaplotypeCallerSpark, with results shown in Fig. 4. The baseline for this experiment is the single-threaded runtime of GATK. The speedup ratios in the figure were calculated by dividing the single-threaded execution time by the runtimes of other methods.

LPA demonstrated better scalability than the other two methods, achieving 30x–40x speedup at 64 threads. HaplotypeCallerSpark showed the worst scalability due to its inability to handle long-running tasks, which significantly limited its performance. While ADS-HCSpark maintained high scalability with fewer threads, its prediction inaccuracy and inability to execute bottleneck tasks immediately limited its scalability in high-thread environments.

### Effectiveness of component

#### Predict accuracy

To evaluate the accuracy of model in predicting runtime, we collected the prediction results from logs. Since our approach is based on the ADS-HCSpark framework, which also relies on prediction, we compared the accuracy of our model with that of ADS-HCSpark. The comparison metrics include the accuracy of predicting the top 1%, top5, and top 5% longest runtime, where the top 1% and top 5 have the greatest impact on the results, while the top 5% also has a significant effect. The comparison results are shown in Table 6. "dataset" represents the dataset, "methods" represents the method, "sum" indicates the total number of predictions made by this method, and "top 1%", "top 5", and "top 5%" represent the number (percentage) of predictions successfully made by the method for the top 1%, top 5, and top 5% longest runtimes, respectively.

The results show that LPA has a high accuracy in predicting the top 1% and top 5, whereas ADS-HCSpark demonstrates suboptimal accuracy in certain datasets. This aligns with the performance results, where ADS-HCSpark underperformed in HG00101 and NA18561, both of which had lower top 1% and top 5 accuracy compared to LPA. Notably, both methods show low top 5% accuracy for HG00281. Upon analysis, we found that the runtime ranks from 9 to 216 in HG00281 fall within the 300–500 s range, accounting for 57.98% of the total. This dense distribution poses a high precision requirement for predictions, which neither LPA nor ADS-HCSpark currently achieves. However, such a distribution also reduces the requirement for prediction accuracy. For example, predicting only the top8 is sufficient to achieve good performance in HG00281. This explains why both LPA and ADS-HCSpark perform well on this dataset. Overall, LPA achieves higher accuracy than ADS while using only two-thirds of ADS-HCSpark’s total predictions.

**Table 6:** Accuracy comparison of LPA and ADS-HCSpark

Dataset	Methods	Sum	Top		
			Top1%	Top5	Top5%
HG00101	LPA	45	5(100%)	5(100%)	24(96%)
	ADS-HCSpark	70	4(80%)	4(80%)	20(80%)
HG00281	LPA	33	3(100%)	4(80%)	9(50%)
	ADS-HCSpark	58	3(100%)	4(80%)	7(39%)
HG01256	LPA	40	4(100%)	5(100%)	23(100%)
	ADS-HCSpark	70	3(75%)	4(80%)	19(82%)
NA12878	LPA	30	3(100%)	5(100%)	18(86%)
	ADS-HCSpark	45	3(100%)	5(100%)	18(86%)
NA18561	LPA	40	4(100%)	5(100%)	22(100%)
	ADS-HCSpark	60	2(50%)	3(60%)	16(73%)

#### Performance of scheduling

**Fig. 5:**
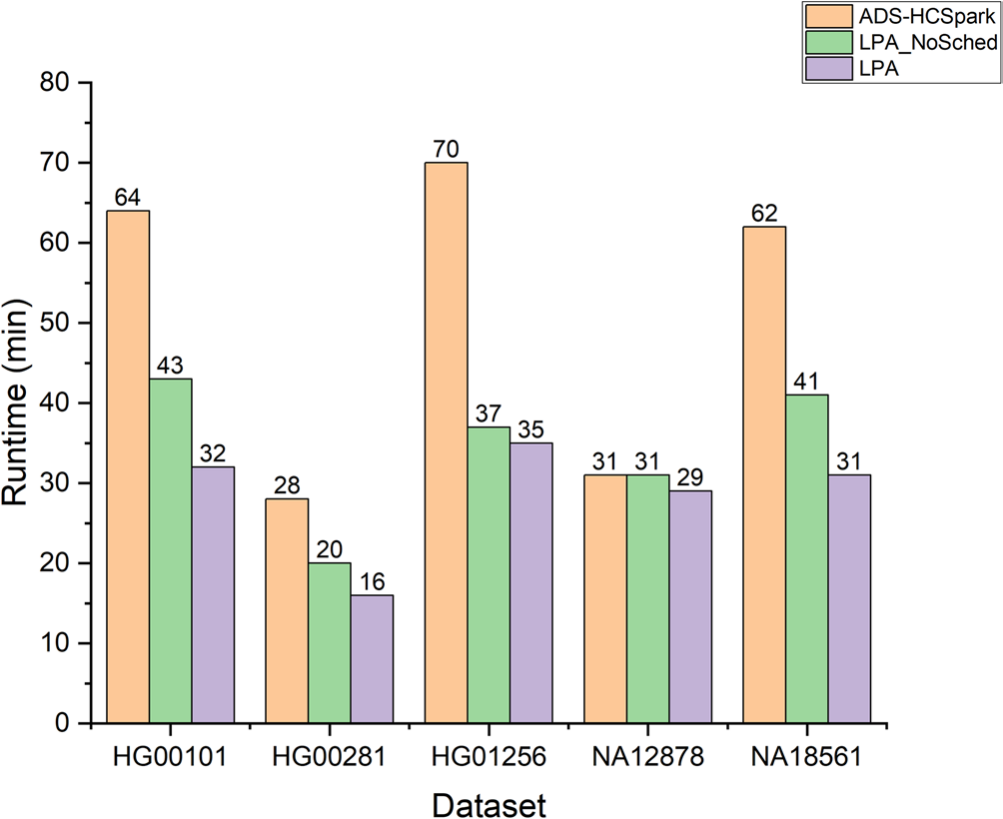
Comparison of ADS-HCSpark, LPA without schedule and entire LPA

To evaluate the performance of the scheduling in LPA, we designed the following experiment involving three methods: ADS-HCSpark, LPA without the scheduling functionality, and the entire LPA. The experimental results are shown in Fig. 5. From the prediction accuracy experiments, we know that LPA outperforms ADS-HCSpark in prediction accuracy. Consequently, LPA without the scheduling functionality clearly outperforms ADS-HCSpark in terms of performance. Furthermore, the complete LPA achieves better performance compared to LPA without scheduling. This is because, based on accurate predictions of each task’s runtime, scheduling can prioritize the execution of long-running tasks, thereby significantly reducing the overall execution time. However, on the NA12878 dataset, there is no difference across the three methods, as all are constrained by a single task with a runtime of approximately 30 min.

#### Results’ accuracy and adaptive splitting performance

To determine the optimal number of splits for long-running data blocks, we conducted the following experiment. In this experiment, long-running data blocks were split into varying numbers of smaller blocks, and their execution times and variant detection accuracy were recorded. Since our method parallelizes HaplotypeCaller, we compared the experimental results with those from single-threaded runs. During the analysis, low-quality variants with a quality score below 30 were excluded. The experimental results, as shown in Table 7, and the best performance data are emphasized using bold font, indicate that splitting data into 6 or 8 blocks significantly enhances performance. While fewer splits result in slightly higher accuracy, the overall impact of the number of splits on accuracy is minimal. This finding aligns with our expectations. According to the results obtained from running on more datasets, we ultimately chose to set the number of splits to 8 to achieve a balance between significantly improved performance and maintaining high accuracy.

**Table 7:** Datasets and metrics comparison

Dataset	Metric	n-Value			
		4	6	8	10
HG00101	Runtime	33.06	33.18	**32.66**	32.75
	Accuracy	**99.93548%**	99.93539%	99.93545%	99.93539%
HG00281	Runtime	17.23	17.63	**16.88**	17.36
	Accuracy	99.95846%	99.95846%	99.95846%	99.95846%
HG01256	Runtime	35.71	**35.45**	35.50	35.48
	Accuracy	**99.92727%**	99.92718%	99.92721%	99.92707%
NA12878	Runtime	29.65	**28.55**	29.46	29.10
	Accuracy	99.91931%	**99.91951%**	99.91948%	99.91938%
NA18561	Runtime	30.86	**30.85**	31.11	31.20
	Accuracy	99.94516%	99.94516%	99.94516%	99.94516%

### System metrics

#### Resource utilization

**Fig. 6:**
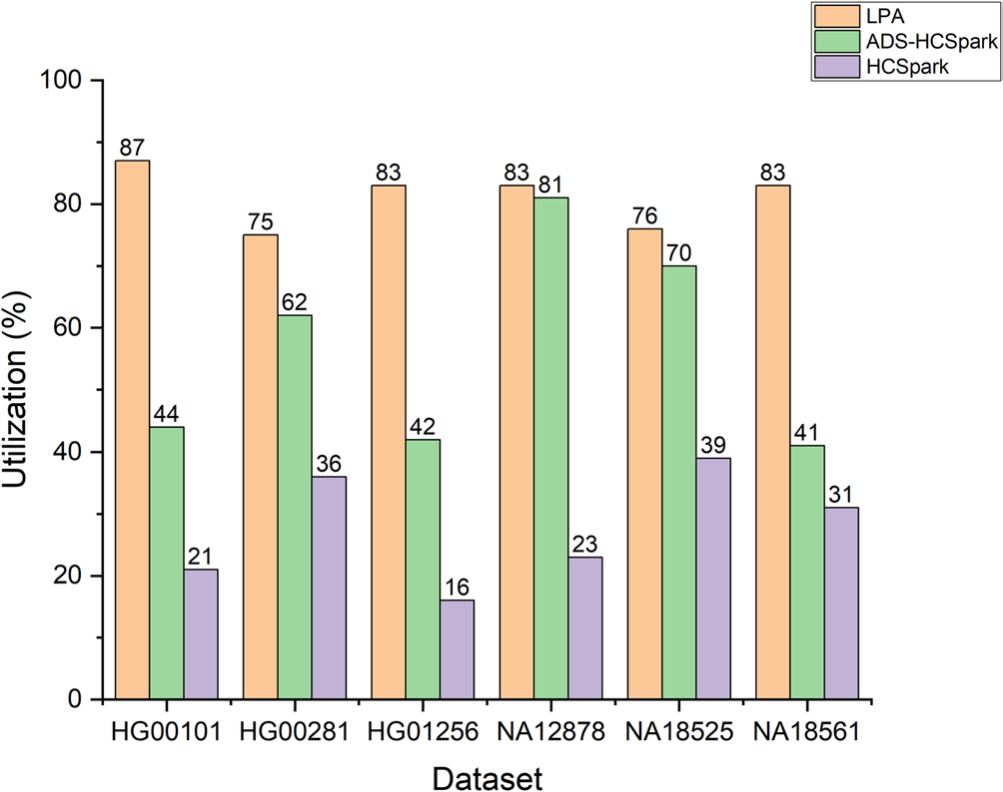
Comparison of LPA, ADS-HCSpark, and HCSpark in CPU utilization

Running HaplotypeCaller requires significant computational resources. And efficient resource utilization is key to reducing costs. Therefore, we evaluated the CPU utilization of LPA during its execution. The results, as shown in Fig. 6, indicate that LPA maintained a CPU utilization rate of over 75% throughout its execution, significantly higher than other methods. This demonstrates that LPA effectively utilizes computational resources, reduces resource waste, and lowers the costs of running HaplotypeCaller.

#### Other system metrics

We also conducted experiments on other system metrics, including memory usage, disk I/O, and network throughput. Among these, disk I/O and network throughput performed as expected: reading BAM files and reference genome data constituted the majority of disk reads, while disk writes were primarily for persisting results, with no additional I/O overhead observed. Network throughput remained low, averaging only 100KB to 300KB per second, which included network traffic from other applications on the server.

**Fig. 7:**
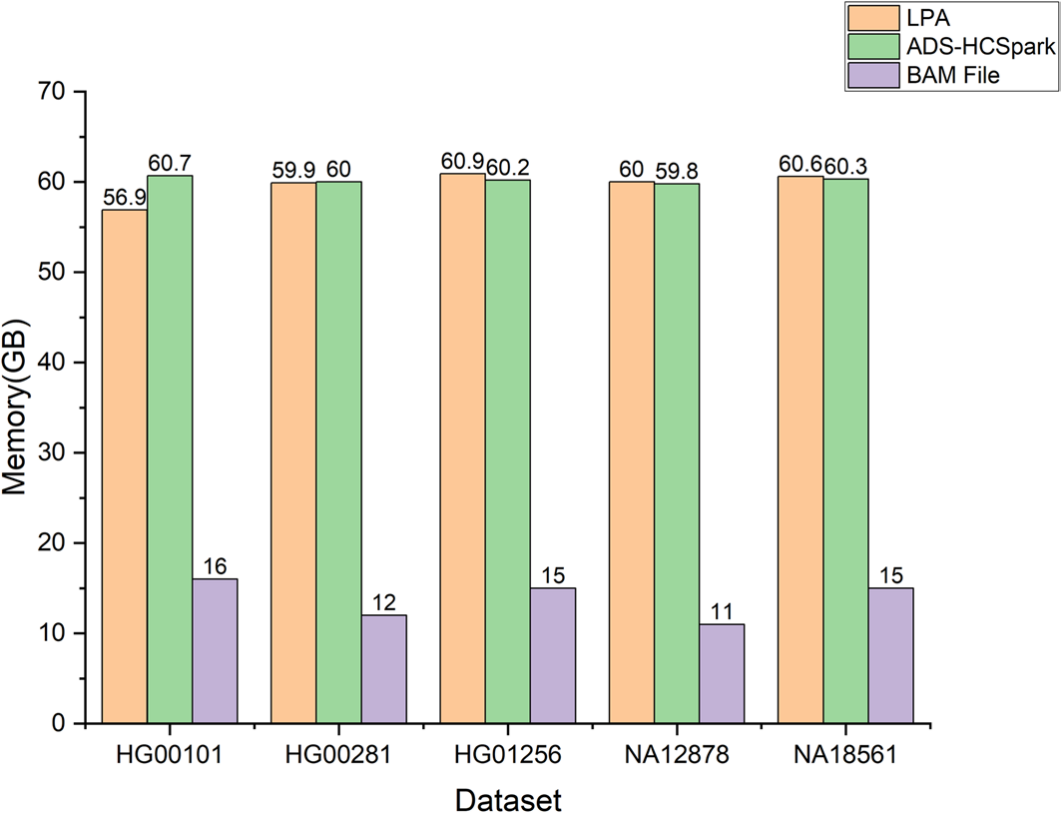
Memory usage of two methods

Notably, memory usage deserves special attention, as shown in Fig. 7, memory occupation of two methods reached a maximum memory usage from 55 to 65 GB, far exceeding our expectations. Since the size of BAM files as shown in Fig. 7, is approximately 10 to 20 GB, the source of the remaining memory consumption remains unclear. This requires us to deeply analyze memory usage details and optimize memory occupation, which will be the focus of our subsequent work.

## Discussion

### Computational skew

Computational skew remains a significant and challenging issue in the parallelization of HaplotypeCaller. Previous methods were unable to accurately predict computational complexity, making it difficult to effectively address this problem. In this work, we introduce AI model to precisely predict computational complexity, enabling us to split high-complexity data blocks, which significantly alleviates computational skew. However, this issue is not fully resolved. For instance, in the HG00281 and NA12878 datasets used in our experiments, although the blocks with the highest computational complexity were accurately identified and split, they still had the longest runtime, becoming performance bottlenecks and limiting overall performance improvements. We also attempted to further split these high-complexity blocks into smaller ones, but the results showed a decline in both performance and accuracy. Developing more effective strategies to handle these extremely high-complexity data blocks and ensure their runtime remains within a reasonable range remains a key challenge.

### High-coverage data

When processing high-coverage datasets, although LPA demonstrated superior performance compared to other similar methods, there is still room for further optimization. In these datasets, the computational complexity of different data blocks is concentrated within a very narrow range, imposing stringent demands on prediction accuracy. Additionally, due to memory limitations, high-coverage data was not fully included during model training, resulting in suboptimal prediction accuracy. This led to negative impact on both performance and resource utilization. In future work, we aim to further optimize the model training process, incorporating more high-coverage data samples to enhance prediction accuracy and fully exploit computational resources.

### Non-human targets

Constrained by the research timeline, this study exclusively utilized human genomic data for model training and evaluation, leaving the non-human generalization capability of the LPA algorithm unexplored. Notably, human genetic data constitutes only a minuscule proportion of the global biological sequencing repository. Under the current framework, LPA’s species-specific modeling mechanism necessitates pre-collection of target species’ datasets, followed by processing these data with GATK to obtain runtime metrics, which are then used for training. This species-dependent modeling paradigm reveals critical limitations in biodiversity research—specifically, high data acquisition costs and prolonged training runtime—where widespread species coverage is required. Achieving universal genomic analysis across all species would demand exponentially increasing data scales and time investments.

This underscores a key challenge for future research: enhancing LPA’s cross-species generalization capability. Our upcoming work will focus on developing strategies to improve the algorithm’s predictive power for unknown species’ genomic data, and maintain high prediction accuracy.

## Conclusion

In this paper, we proposed learning-based framework LPA, a parallel optimization method for HaplotypeCaller that, for the first time, uses model to predict the computational complexity of HaplotypeCaller. By employing adaptive splitting and MKP based task scheduling, we addressed the issue of computational skew. The model achieved significantly higher prediction accuracy compared to other approaches. In terms of performance, LPA demonstrated a 30x–40x improvement over HaplotypeCaller, a 1.3-2x improvement over the similar parallel method ADS-HCSpark on most datasets, and a 2x–5x improvement over HaplotypeCallerSpark. Moreover, LPA maintained over 75% CPU utilization throughout its execution, far exceeding other methods, and maintaining an accuracy with over 99.9%.

## Additional file

Supplementary file 1 (pdf 195 KB)

## Abbreviations

GATK: Genome analysis toolkit
SNP: Single nucleotide polymorphisms
Indels: Insertions/deletions
LPA: Learning-based parallel acceleration
MKP: Multi-Knapsack problem
NGS: Next-generation sequencing
GPU: Graphics processing unit
FPGA: Field-programmable gate array
GBDT: Gradient boosting decision trees
DP: Dynamic programming
