# Supplementary material for: Learning-based parallel acceleration for HaplotypeCaller
Source: BMC Bioinformatics. 2025 Aug 20;26:217. doi: 10.1186/s12859-025-06242-w (PMC12366067; doi:10.1186/s12859-025-06242-w)
Supplement: Supplementary file 1 — (pdf 195 KB) [file 12859_2025_6242_MOESM1_ESM.pdf]

Software Details and Open-Source Availability

The primary tools used in this study include:

- Apache Spark 3.4.3 (https://spark.apache.org/)
- ADS-HCSpark (open-source repository: https://github.com/SCUT-CCNL/ADS-HCSpark)
- LPA(open-source repository: https://github.com/laixx9/LPA)

To run LPA, first fill in your own paths for the model path, chromosome mapping file path, and reference genome mapping file in Arguments.java. After running the model training code, three files will be generated. For subsequent steps, please refer to the README.txt of ADS-HCSpark (https://github.com/SCUT-CCNL/ADS-HCSpark/blob/master/README.md).

For source code compilation:

1. First, obtain gatk-protected by running git clone https://github.com/broadgsa/gatk-protected.git.
2. Enter the gatk-protected folder and execute mvn install -P\!queue.
3. Navigate to the LPA folder, run mvn package, and then locate the sparkhc-1.0-SNAPSHOT.jar package in the target directory.

Then is the **preprocess** stage.

The **command** is:

*spark-submit *

*--master local[64] *

*--total-executor-cores 64 *

*--driver-memory 50G *

*--executor-memory 50G *

*--conf spark.hadoop.mapreduce.input.fileinputformat.split.maxsize=33554432 *

*--conf spark.hadoop.mapreduce.input.fileinputformat.split.minsize=33554432 *

*sparkhc-1.0-SNAPSHOT.jar *

*-t BuildPreprocess *

*-i NA18561.alt_bwamem_GRCh38DH.20150718.CHB.low_coverage.bam *

-c conf.prop

Finally is the **process** stage.

The **command** is:

*spark-submit *

*--master local[128] *

*--total-executor-cores 128 *

*--driver-memory 50G *

*--executor-memory 50G *

*--conf spark.hadoop.mapreduce.input.fileinputformat.split.maxsize=33554432 *

*--conf spark.hadoop.mapreduce.input.fileinputformat.split.minsize=33554432 *

*--conf spark.driver.maxResultSize=0 *

*sparkhc-1.0-SNAPSHOT.jar *

*-t HaplotypeCaller *

*-i NA18561.alt_bwamem_GRCh38DH.20150718.CHB.low_coverage.bam *

*-o data_18561.vcf *

*-c conf.prop *

*-p *

-s

The **conf.prop** is:

FASTA_PREFIX=/path/ucsc.hg19 // reference genome path without .fa/.fasta suffix

DBSNP_DB=none

ADDITION_EACH_SPLIT_SIZE=4

Parameter Explanation

1. **--master local[64]**
   - **Function**: Run Spark in local mode with 64 worker threads.
   - **Purpose**: Execute the job on a single machine using multi-threading, suitable for development or small-scale testing. The [64] specifies the number of concurrent threads to use.
2. **--total-executor-cores 64**
   - **Function**: Allocate 64 CPU cores for executors.
   - **Purpose**: In local mode, this parameter aligns with --master local[64] to ensure all specified cores are used for parallel task execution.

3. **--driver-memory 50G**
   - **Function**: Assign 50 gigabytes of memory to the Spark driver process.
   - **Purpose**: The driver coordinates task scheduling and handles result aggregation. Sufficient memory is critical for large-scale data processing and complex task graphs.
4. **--executor-memory 50G**
   - **Function**: Allocate 50 gigabytes of memory to each executor process.
   - **Purpose**: Executors run task computations. High memory is essential for processing large genomic files (e.g., BAM files) and avoiding out-of-memory errors during data shuffling or aggregation.
5. **--conf spark.hadoop.mapreduce.input.fileinputformat.split.maxsize=33554432**
   - **Function**: Set the maximum size of input file splits to 32MB (33554432 bytes).
   - **Purpose**: Control how input data (e.g., BAM files) is partitioned. Smaller splits increase parallelism but may introduce overhead. 32MB is optimized for balancing task granularity and I/O efficiency in genomic data processing.

6. **--conf spark.hadoop.mapreduce.input.fileinputformat.split.minsize=33554432**
   - **Function**: Set the minimum size of input file splits to 32MB.
   - **Purpose**: Ensure all splits are at least 32MB, preventing 碎片化 (fragmentation) of tiny tasks that could degrade performance. This parameter works with maxsize to enforce consistent split sizes.
7. **sparkhc-1.0-SNAPSHOT.jar**
   - **Function**: The main application JAR file for ADS-HCSpark.
   - **Purpose**: Contains the compiled code for the variant calling framework, including preprocessing and analysis modules.
8. **-t** : the tool name of executing. There are three tools: *BuildPreprocess*, *HaplotypeCaller*, and *vcfmerge*.
9. **-i** : the input file path. The input file path should be an HDFS directory.
10. **-o** : the ouput file path. It should be an HDFS directory. If this parameter is not specified, the outpu file path is the same as the input file path by default.
11. **-p** : it represents using the adaptaive data segmentation. if this parameter is set, data preprocessing step need to be perfomed firstly.
12. **-pf** : the preprocessing file path. When executing ADS-HC, configuring this parameter to indicate the path of preprocessing file. If it is not specified, the default preprocessing file path is the same as the input

file path. The name of preprocessing file is the input file name plus the ".hcidx" suffix.

13. **-s** : sorting and merging into a VCF file. When the merged file is too large, the parameter `spark.dirver.maxResultSize` should be adjusted appropriately.
14. **-dc** : Cache optimization strategy. It is recommended to use when dbSNP is need.
15. **-c** : specify a property file.
